# Asymmetric Expansion with a Modified Quad Helix for Treatment of Isolated Crossbite

**DOI:** 10.1155/2017/7275846

**Published:** 2017-05-14

**Authors:** Marisabel Magnifico, Alberto Di Blasio, Diana Cassi, Chiara Di Blasio, Mauro Gandolfini

**Affiliations:** ^1^Section of Orthodontics, University Dental Center, Department of Medicine and Surgery, University of Parma, Parma, Italy; ^2^Section of Maxillofacial Surgery, University Hospital of Parma, Parma, Italy

## Abstract

Unilateral posterior crossbite often involves only one tooth, especially upper first molar; in these cases it is never easy to obtain an asymmetrical movement of a molar and a proper planning of the orthodontic device with its anchorage is necessary to avoid arch overexpansion. Thanks to its simplicity and efficacy, the modified Quad Helix here described represents a valid therapeutic tool in cases of isolated posterior crossbite.

## 1. Introduction

Posterior crossbite is a common defect of occlusion seen in orthodontic practice. This anomaly is often part of a broader set of orthodontic problems but can also constitute an isolated defect involving only one tooth.

According to different studies, the prevalence of posterior crossbite varies between 6% and 23%. The most frequent is unilateral crossbite, approximately 6-7%, compared to bilateral crossbites, with prevalence between 1.5% and 3.5% [1, 2].

The aetiology of posterior crossbite includes genetics, environmental and functional factors, and habits [3, 4]. Unilateral posterior crossbite can be defined either functional or true unilateral posterior crossbite. True unilateral posterior crossbite can be distinguished from functional crossbite by observing the mandibular path of closure and by determining a crossbite in both centric relation and centric occlusion without a functional shift [2].

However, bilateral crossbite and functional crossbite are usually associated with transverse maxillary deficiency [5, 6]. This deficiency is often the result of asymmetric growth of the maxilla or the mandible, discrepant width of maxilla and mandible, crowding, premature loss, or prolonged retention of primary teeth, impaired nasal breathing, finer sucking, and abnormal swallowing habits [7].

It is well known that not only the anatomic integrity of orofacial structures [8] but also a correct functional dynamics is indispensable for a harmonious growth of the mandible [9]. The crossbite affects these parameters and can create, if not corrected, an asymmetrical growth environment with morphological consequences producing aesthetic discomfort in childhood [10] or leading to complex surgical corrections in adulthood [11].

Treatment options for posterior crossbite correction include maxillary arch expansion, removal of occlusal interferences, and elimination of functional shift. Early crossbite corrections lead to a stable and normal occlusion pattern and contribute to symmetrical condylar growth, harmonious TMJ movements, and overall growth in the mandible [12, 13].

In true unilateral posterior crossbite, the aim should be to move selected teeth on the constricted side of the maxillary arch. If conventional expansion appliances are used to treat true unilateral posterior crossbite, then the maxillary dental arch will be expanded bilaterally, resulting in undesirable overexpansion of the unaffected side [2].

When the posterior unilateral crossbite involves only one tooth, especially the upper first molar, the therapeutic appliances are different, removable, or fixed.

A removable appliance with finger springs or with jackscrews, sectioned asymmetrically, can be used. Sometimes, the low height of the clinical crowns of molars makes retention difficult and lessens the effective force necessary to reproduce maxillary expansion. Unfortunately, any removable appliance leaves the clinician totally dependent on patient cooperation [14, 15] and presents hygiene problems. Elastic can be attached from the lingual attachments of the maxillary teeth to the buccal attachments of the mandibular teeth. Elastics, like removable appliances, require patient compliance and might extrude the involved teeth with the vertical component of the force. This extrusion is undesirable in vertical growers and in patients with limited overbite.

An alternative treatment is to use fixed palatal maxillary expansion appliances. W-arches and Quad Helix appliances can be modified by changing the length of the arms to include more teeth in the anchorage unit. Fixed lingual arches require less overall treatment time and are cost-effective when compared with removable appliances. In this case report, a modified version of the Quad Helix is presented, which is useful in cases of isolated crossbite.

## 2. Case Presentation

**Figure 1:**
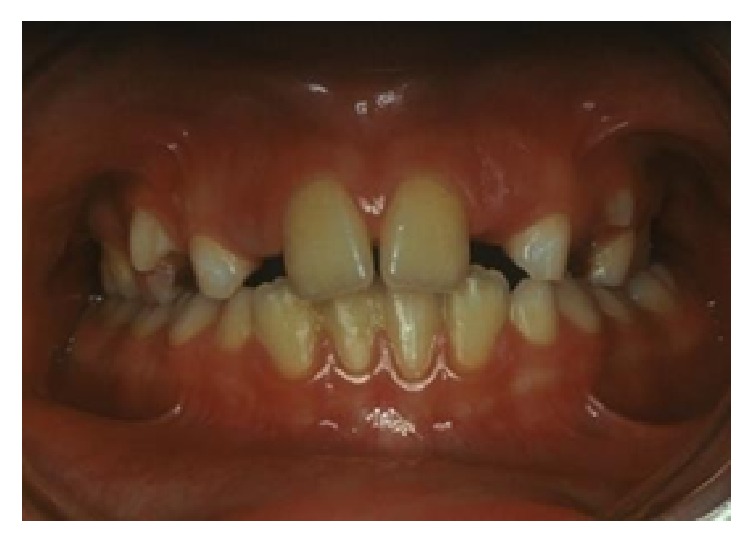
Patient with crossbite of upper left first permanent molar.

An 8-year-old patient comes to our attention for an orthodontic consultation. Clinical and cephalometric [16] evaluations, in compliance with radiation protection criteria especially for growing individuals [17, 18], showed no orthodontic or skeletal problems, except for crossbite of first permanent molar on the left side (Figure 1). The treatment so only consisted in resolution of isolated crossbite and in the control of dental commute over time.

In order to rapidly achieve expansion of the tooth in crossbite, it is recommended to apply a single force capable of determining, in a very short period, the inclination of the tooth to be straightened and subsequent resolution of isolated crossbite.

Thorough knowledge of orthodontic biomechanics allows the operator to manage orthodontic alignment of posterior areas of the arches, thus avoiding in most cases the use of more invasive methods of absolute skeletal anchorage [19].

**Figure 2:**
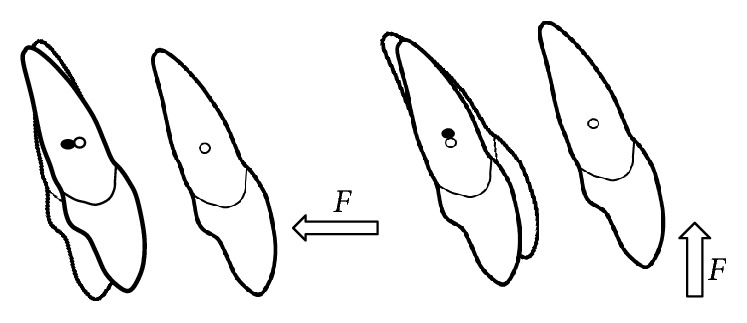
A single force applied to the bracket (not through the center of resistance) will cause rotation of the tooth.

A single point force applied to a tooth has both a magnitude and a direction. When a single force is directed through the center of resistance (*C*_res_), the tooth feels a tendency to translate or to displace all points on the tooth the same amount in the same direction of the applied force. Commonly, a single point force cannot be applied to act directly through *C*_res_ and must be applied at the bracket. When a force does not act through *C*_res_ of a tooth, the tooth rotates (Figure 2).

**Figure 3:**
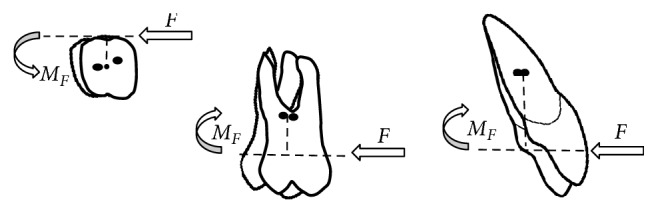
Moment (*M*) produced by a force not acting through *C*_res_.

The rotational tendency, or moment, produced by a force not acting through *C*_res_, is expressed as the moment of the force (*M*). The magnitude of *M* (Figure 3) is measured as the magnitude of the force (*F*) multiplied by the perpendicular distance (*d*) between the line of the force and *C*_res_ (*M* = *F∗d*) [20].

When a true posterior unilateral crossbite involves only one tooth, especially the upper first molar, the design of Quad Helix needs to be modified by leaving the inner wire bordering the palatal faces of the upper premolars and canine on the side that is not to be expanded, as anchorage, and removing the inner wire on the side to be expanded [1].

The single force applied to the single molar generates an expansive force on the lateral arm; this expansive tendency must be balanced by force distribution on more dental units, generally in number of three or four.

The distribution of the expansive force on more teeth, on the unaffected side, avoids having to balance this force with a negative torque, as would happen with the use of palatal bar [21]. Furthermore, this avoids intrusive forces on the anchor molar (and extrusive ones on the side to be expanded) during the correction of the crossbite.

Insufficient upper transverse dentoalveolar compensation often arises with the appearance of unilateral posterior crossbite, characterized by a palatoversion of a single tooth of the upper dental arch.

In 1975 Ricketts describes the Quad Helix appliance [22] that is one of the most versatile appliances that can be used in the early and mixed dentition, because it is easy to use and well tolerated by patients [22].

**Figure 4:**
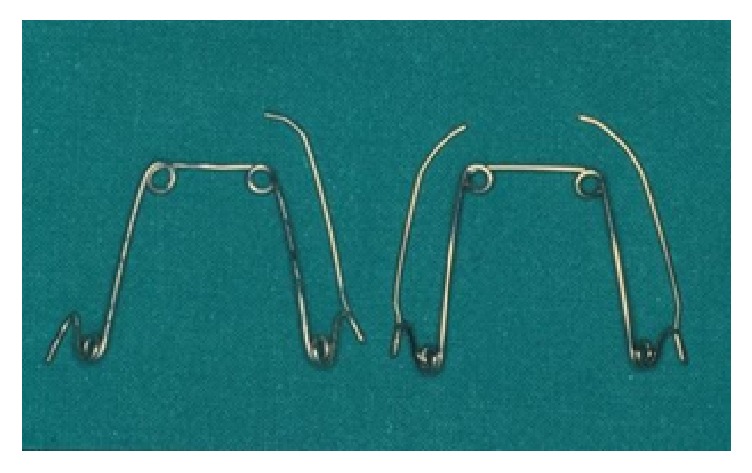
Asymmetric Quad Helix (left) and traditional Quad Helix (right).

Asymmetric Quad Helix is a variant characterized by the presence of a normal lateral arm and double- end terminal only on the anchorage side and, on the active side, of a single wire terminal instead of the double-end one (Figure 4).

**Figure 5:**
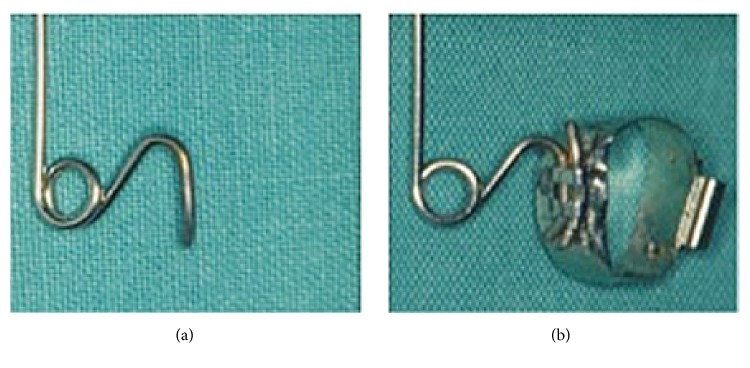
Details of the single terminal (a) and the terminal introduced together with the wire segment of the same diameter into the Goshgarian tube and welded to the band (b).

This single terminal bent back on itself distally in a gingival direction in order to avoid disinsertion. Moreover, it is necessary to place in the palatal attachment that is welded to the band, together with the insert, a segment of wire of the same diameter of the Quad Helix (0.9 mm), the ends of which protrude outwards from the attachment itself and must be appropriately bent in order to avoid disconnection; this avoids the insert from placing itself obliquely which would reduce control of the direction of the applied force (Figure 5).

**Figure 6:**
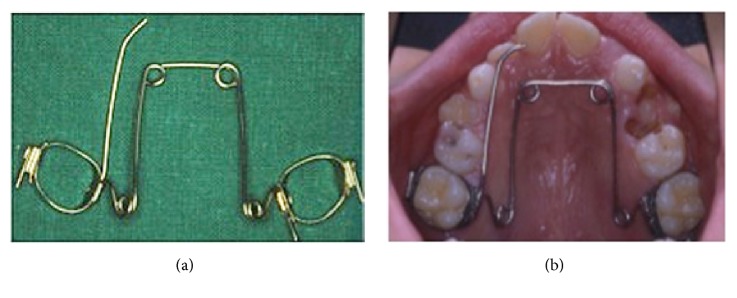
Asymmetric Quad Helix: extraoral (a) and intraoral (b) views. When the auxiliary wire segment is applied into the mouth, it must not interfere either with the mucosa or with the band in order to not give rise to undesired positions or stress.

Moreover, it is necessary to heat both the wire segment and the final millimetres of the insert with a flame in order to blunt the steel, before introducing them into the attachment: this makes bending easier in order to block the position after insertion and also to straighten them more easily when the time comes to take the appliance out. A further fundamental precaution consists in bending the terminal part of the insert so that it is sufficiently separated from the crown of the banded tooth, so that with the expression of the expansive action it does not contact with the crown (Figure 6). In fact, this would induce an undesired couple of forces.

In order to obtain a single expansive force, one could place the single terminal into a simple tube so as not to block it with a segment of auxiliary wire; however, the use of Goshgarian attachments is to be preferred in the eventuality of the possible future insertion of a traditional Quad Helix to improve tooth movement, to continue possible orthodontic treatment on the remaining dental arch, or to ensure retention [23].

**Figure 7:**
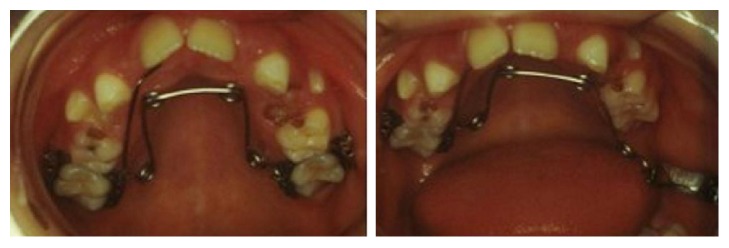
Application and activation system of the asymmetric Quad Helix; it can be seen that there is the absence of a torque control allowing the expression of a single force on tooth 2.6.

**Figure 8:**
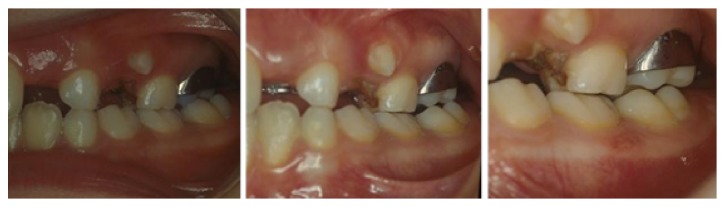
Rapid resolution of crossbite of tooth 2.6.

Activation of the asymmetric QH was equivalent to half the transversal width of the banded molars (Figure 7); crossbite correction was achieved rapidly (1 month along) (Figure 8).

## 3. Discussion

Thanks to its simplicity and efficacy, the modified Quad Helix here described is easy to fabricate, versatile, and useful to resolve an isolated crossbite. The advantages of this modified appliance are significant and include simple design, easy construction, minimal cost, and better results.

In fact, it is never simple to obtain an orthodontic asymmetric movement of a molar, also due to the spatial position that the molars occupy in the oral cavity, being close to gums that can be damaged by bulky orthodontic appliances. In addition, a simpler device will be easier to control, with less costs and less time to care, and therefore much more tolerated by the patient.
